# Unlocking the epigenetic code: new insights into triple-negative breast cancer

**DOI:** 10.3389/fonc.2024.1499950

**Published:** 2024-12-18

**Authors:** Gowthami Mahendran, Ann Dharshika Shangaradas, Ricardo Romero-Moreno, Nadeeshika Wickramarachchige Dona, S. H. G. Sumudu Sarasija, Sumeth Perera, Gayathri N. Silva

**Affiliations:** ^1^ Department of Chemistry, Faculty of Science, University of Colombo, Colombo, Sri Lanka; ^2^ Harper Cancer Research Institute, South Bend, IN, United States; ^3^ Department of Biochemistry, Faculty of Medicine, Sabaragamuwa University of Sri Lanka, Ratnapura, Sri Lanka

**Keywords:** triple-negative breast cancer (TNBC), epigenetic modifications, DNA methylation, histone deacetylase inhibitors (HDACi), microRNA (miRNA)

## Abstract

Triple-negative breast cancer (TNBC) is a highly aggressive and clinically challenging subtype of breast cancer, lacking the expression of estrogen receptor (ER), progesterone receptor (PR), and HER2/neu. The absence of these receptors limits therapeutic options necessitating the exploration of novel treatment strategies. Epigenetic modifications, which include DNA methylation, histone modifications, and microRNA (miRNA) regulation, play a pivotal role in TNBC pathogenesis and represent promising therapeutic targets. This review delves into the therapeutic potential of epigenetic interventions in TNBC, with a focus on DNA methylation, histone modifications, and miRNA therapeutics. We examine the role of DNA methylation in gene silencing within TNBC and the development of DNA methylation inhibitors designed to reactivate silenced tumor suppressor genes. Histone modifications, through histone deacetylation and acetylation in particular, are critical in regulating gene expression. We explore the efficacy of histone deacetylase inhibitors (HDACi), which have shown promise in reversing aberrant histone deacetylation patterns, thereby restoring normal gene function, and suppressing tumor growth. Furthermore, the review highlights the dual role of miRNAs in TNBC as both oncogenes and tumor suppressors and discusses the therapeutic potential of miRNA mimics and inhibitors in modulating these regulatory molecules to inhibit cancer progression. By integrating these epigenetic therapies, we propose a multifaceted approach to target the underlying epigenetic mechanisms that drive TNBC progression. The synergistic use of DNA methylation inhibitors, HDACi, and the miRNA-based therapies offers a promising avenue for personalized treatment strategies, aiming to enhance the clinical outcome for patients with TNBC.

## 1 Introduction

### 1.1 Breast cancer: genetic factors and therapeutic advancements

Breast cancer (BC) is the most commonly diagnosed malignancy in 30% of women each year and the risk to develop BC is known to have a hereditary component with a mean diagnostic age of 62 years with a higher risk among black women (1). Most breast cancers of women start within the ducts or lobes which are identified as ductal carcinoma or lobular carcinoma. These breast cancers which do not extend beyond the milk duct or lobules in the breast are noninvasive. However, invasive breast cancer spread into the neighboring tissues and thereby demonstrate specific molecular features. Some of the genetic causes which contribute to cancer progression are the high-penetrance genes (*BRCA1*, *BRCA2*, *p53*, *PTEN*, *ATM*, *NBS1*, and *LKB1*) (2), cytochrome P450 genes which are low penetrants (*CYP1A1*, *CYP2D6*, and *CYP19*) (3), genes of glutathione S-transferase family (*GSTM1* and *GSTP1*) (4, 5), alcohol and one-carbon metabolism genes (*ADH1C* and *MTHFR*) (6), genes involved in DNA repair (*XRCC1*, *XRCC3*, and *ERCC4/XPF*) (7), and cell signaling molecule encoding genes [*PR*, *ER*, *TNF-α*, or heat shock protein 70 (*HSP70*)] (8, 9). Among these frequently occurring mutations, changes in the *BRCA1* and *BRCA2* (Breast Cancer 1 and 2) genes are present in approximately 80-90% of all hereditary BCs (10), which account for less than 25% of African American women population with TNBC (11). Additionally, other common BC risk factors include lifestyle hormonal influence, socioeconomic background, age, diet, obesity, and radiation exposure (1). Medical advancements have played a crucial role in improving the survival rates of cancer patients. The *HER2* (human epidermal growth factor receptor 2)/neu gene, one of four members of the epidermal growth factor receptor (EGFR) family, was identified as a contributor to the development of breast cancer in patients through the ground-breaking work of William Muller et al. (12) in 1988. Their work laid the foundation for the development of the drug Herceptin (clinical name: trastuzumab), which is a recombinant, humanized monoclonal antibody that selectively binds to the extracellular domain of HER2/neu (12). In combination with paclitaxel, Herceptin was approved by the Food and Drug Administration (FDA) as a first-line treatment of HER2+ metastatic BCs (13).

**Figure 1:**
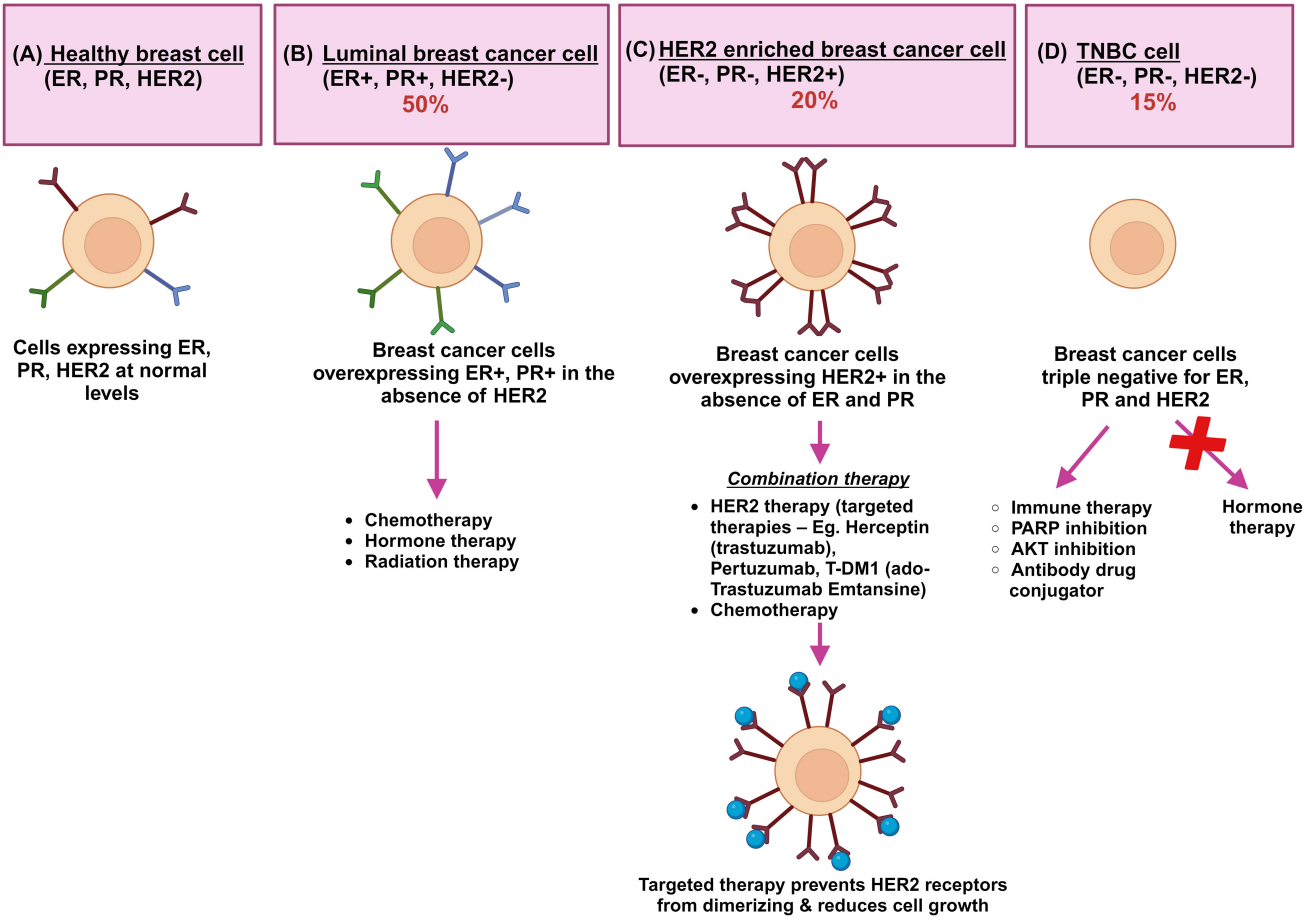
A schematic representation of the key breast cancer subtypes: the schematic shows **(A)** healthy breast cells where ER, PR, and HER2 receptors are normally present. **(B)** The luminal breast cancers of A and B types are represented together where normal levels of ER and PR expression and low or no expression of HER2 protein (ER+, PR+, HER2-) are found. Hormonal therapy is effective and Ki67 immunohistochemical marker is critically important in differentiating A and B subtypes. **(C)** HER2 enriched breast cancers (ER-, PR-, HER2+) overexpress ERBB2 receptors which contain receptor-independent tyrosine kinase domains. Homodimerization and heterodimerization of HER2 receptors contribute to tumor development and progression. Drug treatment including Herceptin (Trastuzumab), trastuzumab combined with emtasin (T-DM1), pertuzumab, and tyrosine kinase inhibitors are used as targeted therapy. Herceptin, a humanized immunoglobulin G1 antibody, blocks the ligand-independent activation of HER2+ cells. **(D)** Triple negative breast cancers (ER-, PR-, HER2-) lack expression of all three receptors and are resistant to standard hormonal therapies. Alternative therapies are being tested currently for TNBC. ER is green, PR is blue, and HER2 is maroon; Herceptin immunotherapy is shown as blue colored circles (14).

As BC is increasingly recognized as a heterogeneous cancer type, it is characterized by significant variations in genomic and transcriptomic profiles. Based on pathological, immunohistochemical and molecular features, invasive BC has been classified into luminal A, luminal B, HER2 and triple negative A and triple negative B subtypes (**Figure 1**) (15). A further study by Dai et al. (16), using breast cancer cell lines, has demonstrated how luminal A and B cell lines are distinguished from one another, while HER2-positive lines are grouped as a single subtype to support studies on tissue subtyping and drug response experiments targeting ER and/or HER2. Triple-negative lines are divided into two separate groups, corresponding to basal A (BL1 and BL2 subtypes) and B (M and MSL subtypes). Within the broader spectrum of BC, triple-negative breast cancer (TNBC) represents an aggressive and distinct subtype, characterized by the absence of ER, PR, and HER2 expression, making it more challenging to treat and often associated with poorer prognosis.

## 2 TNBC: molecular subtypes, therapeutic targets, and emerging treatment strategies

TNBC is a heterogeneous type of BC that comprises nearly 15-20% of all diagnosed cases (17, 18). As the name suggests, TNBC is HER2, ER and PR negative, rendering it resistant to therapeutics designed to target these receptors. Among the BC diagnosis, TNBCs distinctive nature of its molecular profile and the metastatic patterns often involves the brain and lungs (less likely to bones), which is exceptional from other subtypes of BCs (19, 20).

TNBC subtyping is concerned, Lehmann et al (21), using microarray-based gene expression profiling, have categorized it into six distinct molecular subtypes: Basal-Like 1 (BL-1), Basal-Like 2, Immunomodulatory (IM), Mesenchymal (M), Mesenchymal Stem-Like (MSL) and Luminal Androgen Receptor (LAR). Subsequently, Lee et al. classified TNBC into four main subtypes: BL-1, BL-2, M and LAR (22). In recent years, molecular profiling has led to the identification of several distinct subtypes within TNBC (23, 24). Most of the TNBC is represented by basal-like subtypes (around 75%). BL-1 and BL-2 subtypes are distinguished by their association with cell cycle pathways and growth factor signaling pathways, respectively. The LAR subtype, a steroid hormone class, belonging to ER and PR receptors, represent 15-20% of all BCs (25). M and MSL subtypes are involved in cell motility and differentiation. IM type influences the immune cell processes, which can also be considered as another type of BL. More recently, a study by Hu et al. demonstrated an immunohistochemistry (IHC) surrogate classification as a practical and desirable approach (26). Through differential expression analysis, mRNA/protein correlation, and Receiver operating characteristic (ROC) analysis, a surrogate classification scheme for TNBC was proposed, using androgen receptor (AR), CD8, Forkhead box C1 protein (FOXC1), and doublecortin-like kinase 1 (DCLK1). This scheme had significant alignment with gene expression subtypes reported in literatures before and can help in TNBC patient prognosis (26).

Around 15% of the BC patients accounting for TNBC exhibit a more aggressive clinical profile than any other cancer type. The heterogenicity of TNBC makes it more vulnerable to chemotherapy than any other BCs. However, the number of TNBC subtypes that occur in women remains a subject of ongoing investigation (27, 28). Furthermore, TNBC patients exhibit elevated expression levels of CK5 (cytokeratin 5), CK14, caveolin-1, carbonic anhydrase IX (caix), p63, EGFR/HER1 with DNA repair proteins, while BRCA1/2 mutations are also observed (29). Neoadjuvant and adjuvant therapeutic strategies are frequently employed in the treatment of TNBC. While Carboplatin adjuvant therapy tends to increase treatment-based positive outcome of TNBC, adjuvant therapy with anthracycline- and taxane-based regimens offers the most significant benefits to TNBC patients as it can decrease the chance of breast cancer recurrence, lower breast cancer-related deaths, and reduce overall mortality (27). Carboplatin enhances pathological complete remission which refers to the absence of the invasive cancer in the breast and thereby increases the survival when added to the neoadjuvant chemotherapy (30). Multiple studies have demonstrated that incorporating platinum or targeted agents like bevacizumab (Bev) (25), Poly ADP-ribose polymerase 1 inhibitors (PARPi), and PD-1/PD-L1 inhibitors (Pembrolizumab, Atezolizumab, Durvalumab, Nivolumab) into standard neoadjuvant treatments enhances pathological complete response rates in TNBC. The global acceptance of PARPi in clinical settings for TNBC treatments is due to its indispensable significance in ensuring the safety and tolerance in patients (31). Either as a monotherapy or as a combination therapy with chemotherapy, progression-free survival (PFS), overall survival (OS), overall response rate (ORR) was measured in TNBC patients. Findings revealed improved PFS and OS and high ORR, although increased risk of grade 3–4 thrombocytopenia (low platelet counts) was observed (32). Hence, monotherapy was lower riskier than the combination therapy in terms of disease progression and higher ORR.

Moreover, studies have also shown that combination of PI3K and CDK4/6 inhibitors to be effective in reducing early adaptation responses to single-agent treatments, helping to overcome resistance in ER-positive breast cancers (33, 34). In a similar vein, another study demonstrated that the combined inhibition of PI3Kα and CDK4/6 is synergistically effective in TNBC models (35). The treatment of ribociclib and BYL719 alongside an immune checkpoint inhibitor (ICI) achieved total tumor regression in xenograft models of TNBC (36, 37). However, elucidation from recent studies portrays the potential role of platinum agents in both neoadjuvant and metastatic settings.

PARP inhibitors have been an effective strategy to treat double-stranded DNA breaks by homologous recombination (27, 28). Olaparib, Velaparib and PF-01367338 are some of the PARP inhibitors that are currently in clinical trials (38, 39). Clinical trials investigating PARP inhibitors in TNBC have yielded encouraging outcomes, notably in patients with BRCA mutations. These studies have exhibited notable enhancements in PFS and overall response rates among TNBC patients treated with PARP inhibitors compared to conventional chemotherapy regimens. Additionally, PARP inhibitors have demonstrated favorable safety profiles, with manageable adverse effects. Olaparib is one of the prominent PARP inhibitors that has been extensively evaluated in TNBC. In a phase III clinical trial, known as the OlympiAD trial, olaparib exhibited superior PFS in comparison to standard chemotherapy among patients with HER2-negative metastatic breast cancer and a germline BRCA mutation (40). Subsequent analyses focusing specifically on the TNBC subset showcased even more pronounced benefits, with significantly prolonged PFS observed in patients treated with olaparib versus chemotherapy. However, despite these encouraging findings, not all TNBC patients exhibit responsiveness to PARP inhibitor therapy. Resistance mechanisms, including the restoration of homologous recombination DNA repair pathways, can curtail the efficacy of PARP inhibitors. Ongoing research endeavors aim to decipher these resistance mechanisms and develop strategies to circumvent them. These include exploring combination therapies targeting alternative DNA repair pathways or molecular alterations that sensitize tumors to PARP inhibition (41). On the other hand, Chimeric antigen receptor T-cell (CAR-T) therapy is another emerging yet a promising treatment for TNBC, especially considering the limitations associated with traditional therapies. Recent studies have demonstrated the potential of CAR-T cells designed to target specific antigens that are overexpressed in TNBC, such as EGFR and HER2. For example, a clinical trial conducted in 2023 found that HER2-targeted CAR-T therapy significantly reduced tumor burden in patients with metastatic TNBC, yielding encouraging response rates (42). Furthermore, advancements aimed at enhancing CAR-T cell persistence and minimizing toxicity have improved the therapeutic index, making this approach more viable for TNBC patients. Nonetheless, challenges persist, including the need to identify suitable tumor-specific antigens and address the immunosuppressive nature of the tumor microenvironment (TME), which can impede CAR-T effectiveness (43). Ongoing research is focused on exploring combination strategies, such as pairing CAR-T therapy with immune checkpoint inhibitors, to improve patient outcomes and tackle these challenges in TNBC treatment. While CAR-T therapy represents a novel approach targeting specific tumor antigens in TNBC, Programmed death-ligand 1 (PD-L1) inhibitors offer a complementary strategy by enhancing the immune response against tumor cells through checkpoint blockade, making them both critical components in the evolving landscape of TNBC treatment. PD-L1 inhibitors are increasingly recognized as a valuable treatment option for TNBC, particularly due to the immunogenic characteristics of this subtype. Recent clinical studies have demonstrated the efficacy of PD-L1 inhibitors, such as atezolizumab and pembrolizumab, when used in combination with chemotherapy (44). For instance, the KEYNOTE-355 trial showed that pembrolizumab combined with chemotherapy significantly improved progression-free survival compared to chemotherapy alone in patients with advanced TNBC, with a median progression-free survival of 9.7 months versus 5.6 months for chemotherapy alone (45). Additionally, a 2023 study found that adding atezolizumab to neoadjuvant chemotherapy resulted in a 51% pathological complete response rate among patients with high PD-L1 expression, compared to 30% in those receiving chemotherapy alone (46). Furthermore, ongoing research is focused on identifying biomarkers, such as tumor mutational burden and specific gene expressions, which will predict patient response to PD-L1 inhibitors thereby refining patient selection (47). Despite these promising findings, challenges remain including the management of immune-related adverse events and understanding the mechanisms of resistance to PD-L1 therapy. Future investigations are anticipated to explore combination strategies with other immunotherapies and targeted therapies to enhance the overall effectiveness of PD-L1 inhibition in the treatment of TNBC.

## 3 Epigenetic modifications in TNBC

Epigenetics plays a pivotal role in shaping the phenotype of an organism or cell by modulating gene expression patterns without altering the underlying DNA sequence. Unlike typical genetic changes, which involve alterations in the DNA sequence itself, epigenetic modifications are reversible and can be dynamically influenced by various environmental factors, developmental cues, and disease states (48). This dynamic nature of epigenetic regulation allows cells to adapt to changing environments and respond to internal and external stimuli, ultimately influencing cellular functions and phenotype. Epigenetic modifications, including DNA methylation, histone modifications, and non-coding RNAs, play pivotal roles in regulating gene expression patterns in TNBC cells (49).

However, in cases of epigenetic dysregulation—encompassing changes in DNA methylation, histone modifications, and imbalances in regulatory proteins like the bromodomain and extra-terminal domain protein family (BET proteins)—it plays a vital role in the development and treatment resistance of TNBC. Furthermore, these epigenetic alterations critically influence the TME, affecting immune cell composition, cytokine signaling, and the expression of immune checkpoints, which ultimately leads to immune evasion. Aberrant DNA methylation patterns, often leads to disruption of histone methyltransferases such as EZH2 and HDACs, gene silencing, affecting key tumor suppressor genes, and signaling pathways involved in cell proliferation, apoptosis, and metastasis. For example, in TNBC, hypermethylation of CpG islands within the promoter regions of tumor suppressor genes, such as *BRCA1* and *PTEN*, leads to their transcriptional silencing contributing to tumor initiation and progression (50). Similarly, dysregulated histone modifications, such as histone acetylation and methylation, contribute to the aberrant gene expression profile characteristic of TNBC influencing tumor aggressiveness and therapeutic resistance. Furthermore, non-coding RNAs such as microRNAs and long non-coding RNAs participate in the epigenetic regulation of TNBC by modulating gene expression at the post-transcriptional level.

Moreover, epigenetic modifications are intricately involved in mediating the response to various therapeutic interventions in TNBC. The stability of epigenetic changes, although generally considered reversible, can vary depending on the specific type of modification and the cellular context. While some epigenetic marks may be transient and dynamically regulated, others can exhibit long-term stability and heritability across cell divisions. Understanding the stability of epigenetic changes in TNBC is crucial for predicting treatment response and developing strategies to overcome therapeutic resistance. Furthermore, the interplay between genetic and epigenetic alterations in TNBC underscores the complexity of cancer development and progression. While genetic mutations provide the initial driving force for tumorigenesis, epigenetic changes can further amplify and fine-tune the oncogenic signaling pathways, leading to the emergence of aggressive phenotypes and therapeutic resistance (51). Integrative analyses of genetic and epigenetic landscapes in TNBC have revealed intricate regulatory networks and potential vulnerabilities that can be exploited for targeted therapies. Thus, epigenetics bridges the gap between genotype and phenotype by regulating gene expression patterns in response to environmental cues and cellular signals. In TNBC, aberrant epigenetic alterations contribute to tumorigenesis, metastasis, and therapeutic resistance, highlighting the importance of elucidating the underlying mechanisms and exploiting epigenetic vulnerabilities for precision medicine approaches. Further, ductal carcinoma *in situ* (DCIS), though classified as a stage 0 TNBC, has shown that nearly 20-50% of patients diagnosed with it have progressed to invasive breast cancer after a 30-year illustrating the need for proper detection/diagnosis method (52). Similar to TNBC scenario, detecting triple-negative ductal carcinoma *in situ* (TN-DCIS) is clinically challenging, highlighting the need for strategies to investigate the molecular events that drive the progression from pre-invasive TN-DCIS to invasive TNBC. A comparative methylation analysis by Fleischer et al. of healthy controls, DCIS, and invasive breast cancer tissues, identified significant changes in methylation profiles across different stages of disease progression (53, 54). Notably, most methylation alterations, both increases and decreases, occurred during the transition from healthy breast tissue to DCIS. In contrast, the changes in methylation patterns from DCIS to invasive breast cancer were relatively minor and therefore supports the hypothesis that methylation changes play an early role in the carcinogenesis of breast cancer, making them a promising target for enhancing early diagnosis (55). Hence, understanding the epigenetic alterations driving TNBC and targeting these epigenetic mechanisms in conjunction with immunotherapy, not only sheds light on its molecular mechanisms but also holds promise for the identification of novel therapeutic targets and the development of epigenetic-based therapies to improve patient outcome. The ongoing research efforts continue to unravel the complex interplay between epigenetics and TNBC paving the way for personalized treatment strategies and precision medicine approaches in the management of this aggressive breast cancer subtype. In this backdrop, this article reviews three of the epigenetic modifications: DNA methylation, histone deacetylation, and miRNA-mediated modifications and their involvement in TNBC development.

### 3.1 DNA methylation: a critical epigenetic regulator of TNBC

DNA methylation is one of the critical epigenetic factors involved in the regulation of gene expression and genomic stability and is biologically necessary for the maintenance of many cellular functions (56). It plays a significant role in vital processes such as imprinting, X chromosome inactivation, chromatin organization, repression of repetitive element transcription, etc (57, 58). DNA methylation involves the covalent modification of a cytosine ring at the 5’ position of a CpG dinucleotide by the addition of a methyl group at the fifth carbon of the ring. This process utilizes S-adenosyl methionine (SAM) as a methyl donor, with DNA (cytosine 5) methyltransferases (DNMTs) catalyzing the methylation process (59). The dynamic interplay within the highly conserved mammalian DNMT family, consisting of DNMT1, DNMT2, DNMT3A, DNMT3B, and DNMT3L, is critical for the accurate and flexible control of DNA methylation in mammalian cells (60).

**Figure 2:**
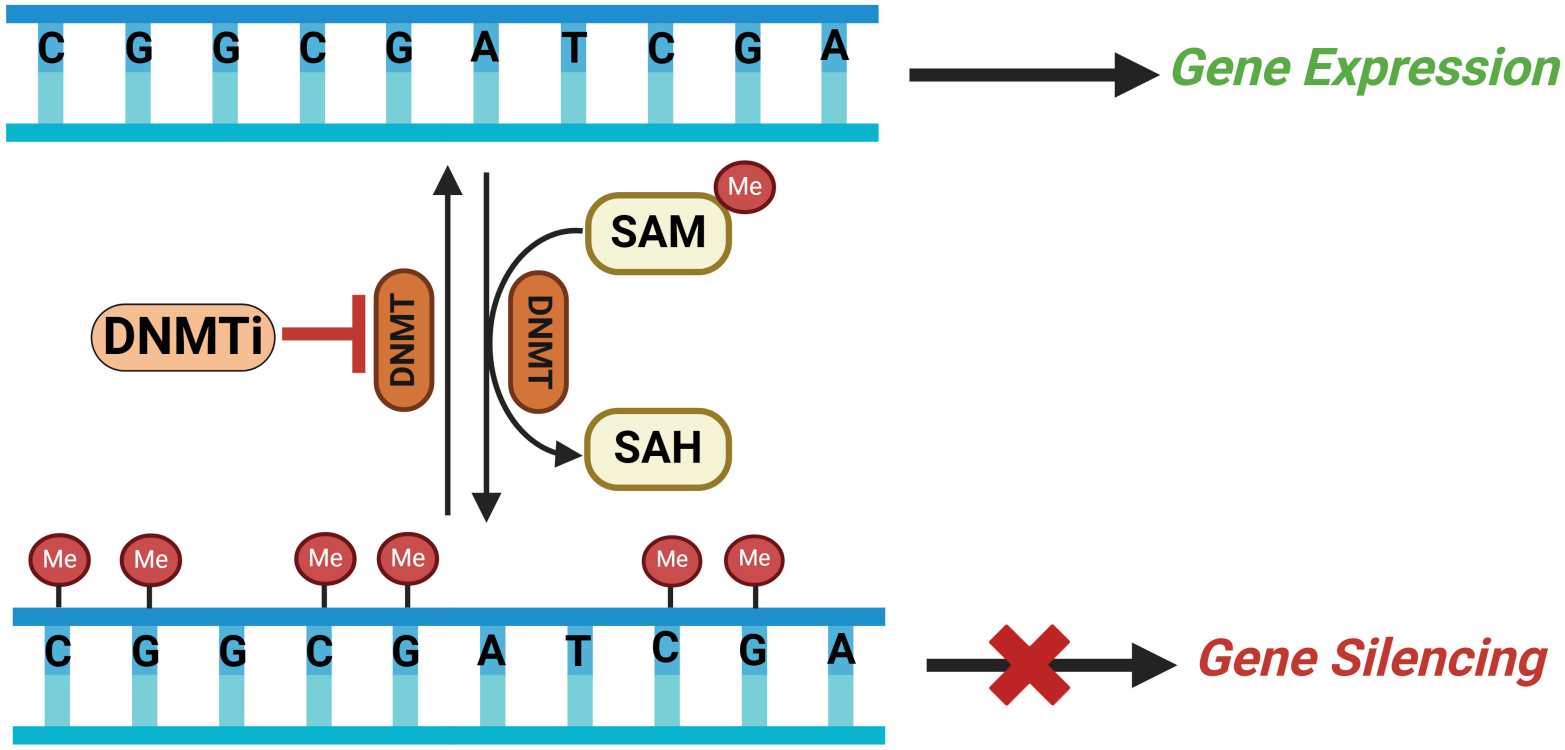
Schematic illustrating the DNA methylation process catalyzed by DNMTs on CpG islands in a gene’s promoter region leading to gene silencing and how DNMT inhibitors (DNMTi) can reverse this methylation resulting in gene expression. SAM, S-adenosylmethionine; SAH, S-adenosylhomocysteine; DNMT, DNA methyltransferaces; DNMTi, DNA methyltraserase inhibitors; Me, methyl group.

As a critical regulator of gene expression, distinct methylation patterns can underlie the onset of various conditions, including cancer (61). These divergent DNA methylation patterns are mainly divided into two types: hypomethylation, characterized by reduced methyl group levels, and hypermethylation, marked by increased methyl group levels. Global hypermethylation usually occurs across the entire genome while hypomethylation takes place at specific sites known as CpG islands (CGIs). The term CpG refers to the base cytosine (C) linked by a phosphate bond to the base Guanine (G) in the DNA nucleotide sequence, which usually clustered together and typically located at or near the promoters and transcription sites of genes (**Figure 2**). Increased level of genome-wide hypomethylation results in increased chromosomal instability and activation of regulatory DNA sequences, including transcription of oncogenes, retrotransposons as well as genes encoding proteins involved in the development of malignant cells (62).

The molecular mechanisms driving CpG hypermethylation in numerous human cancers, including TNBC (63), have been investigated. Frequent hypermethylation of CpG is commonly found in the promoter regions of specific genes including tumor suppressor genes (TSGs) that results in the transcriptional silencing of these genes (64). According to Knudson’s two-hit model hypothesis, the loss of TSG function in cancer arises from the deletion or inactivation of both alleles. TSG mutations are considered recessive and a single mutation in a TSG is typically insufficient to initiate cancer. However, haploinsufficiency of TSGs can result in reduced protein production. Hence, the presence of m5CpG in the promoters of TSGs, which may result in decreased protein production, is regarded as a significant event in TNBC (65, 66). In fact, acquisition of specific patterns of hypermethylation at the CpG islands of certain promoters is frequently observed in many cancer types (67).

There are multiple mechanisms through which CpG methylation can inhibit gene expression. CGI methylation can lead to the binding of methyl-CpG-binding domain (MBD) proteins. These proteins then recruit histone-modifying and/or chromatin-remodeling complexes to the methylated site, inhibiting gene expression by forming a more compact and inactive chromatin structure (66). While DNA methylation can hinder the binding of transcription factors to the promoter, a recent study indicates that such interference is not common (68). Methylation may occur not only in CpG islands but also in CpG shores, which are regions near CpG islands with lower C+G content. This methylation pattern may represent a form of tissue-specific methylation and gene expression regulation (69).

TNBCs is classified into three distinct methylation clusters associated with better or worse prognosis and identified 17 differentially methylated regions (DMRs) that show a strong association with overall survival (OS), including DMRs located in the Wilms tumor 1 (WT1) gene, bi-directional-promoter and antisense WT1-AS (70). BRCA1 promoter methylation is a common occurrence in TNBCs, leading to a tumor phenotype resembling that of BRCA1-mutated tumors (71, 72). Further, methylation of the BRCA1 promoter can affect how sporadic TNBC responds to chemotherapy agents, potentially impacting treatment outcomes. Moreover, detectable methylation of the BRCA1 gene promoter in peripheral blood DNA can serve as a marker for increased susceptibility to TNBC (73). Methylation is a significant mechanism regulating cancer stem cell genes such as CD44, CD133, and Musashi-1, with gene hypomethylation correlating with TNBC (74). Therefore, assessment of epigenetic modifications in breast cancer stem cell (BCSC) genes may offer a more precise TNBC classification and potential therapeutic targets (74). Consequently, DNA methylation emerges as a crucial epigenetic modification, given its well-established role in the malignant transformation of cells through the silencing of essential tumor suppressor genes (52).

Several studies have illustrated that differential methylation of specific CpGs may be useful biomarkers for predicting the response of patient tumors to their treatment (54). All-trans retinoic acid (atRA) regulates gene expression and is used to treat acute promyelocytic leukemia (75). In a study recognizing the critical role of DNA methylation in gene expression regulation, it was hypothesized that differential DNA methylation could predict the response of TNBCs to atRA. To identify biomarkers for the treatment of TNBC with atRA, the effects of atRA on the tumor growth of 13 TNBC cell lines were characterized (76). The findings revealed over 1400 differentially methylated sites in both atRA-resistant and sensitive cell lines. These CpG sites successfully predicted the response of four TNBC patient-derived xenografts to atRA. Subsequently, these xenografts were instrumental in refining the profile, ultimately suggesting that up to 17% of TNBC patients could potentially benefit from atRA treatment (76).

Recent research suggests that TNBC likely has a unique signature, primarily because it lacks three key receptors that set it apart from other types of breast cancer. Additionally, integrating methylation and gene expression into a unified framework may enhance the specificity of detecting TNBC signatures (77). Although TNBC has poor clinical prognosis, it is believed that specific drugs could target the detected TNBC-specific signatures (77). One approach was to coupling methylation and expression changes in TNBC to identify the methylation-regulated signature genes for TNBC. To date, nearly 114 genes with both altered methylation and expression, and 356 existing drugs targeting 10 of the 114 genes have been identified (77). However, it has been revealed that with the ability of BRCA1/2 silencing from hypermethylation origin, the BRCA1 influence can exceed much higher than 25% (11). Due to obesity and other disparities on hypermethylation, the implementation of diet and exercise have been suggested as the starting point for slowing down progression and improving prevention for African Americans with TNBC.

An initial study identified 38 TNBC-specific genes with altered expression compared to normal samples. Later, it was found that the expression of 16/38 TNBC-specific genes were associated with alteration in DNA methylation (50). Novel methylation changes between primary tumors and lymph node metastases, as well as those associated with survival were also identified during the same study. That study revealed the importance of DNA methylation in altered gene expression of TNBC-specific genes (50). The novel insights into progression of TNBC to secondary disease might have provided potential prognostic indicators for this aggressive and hard-to-treat BC subtype. It has also been shown that the TET1 (Ten-eleven translocation) DNA demethylase is specifically overexpressed in about 40% of patients with TNBC, where it is associated with hypomethylation of up to 10% of queried CpG sites and a worse overall survival (78, 79). A connection of TET1 to hypomethylation and activation of cancer-specific oncogenic pathways, including PI3K, EGFR, and PDGF was also found. TET1 expression correlated with sensitivity to drugs targeting the PI3K–mTOR pathway, and CRISPR-mediated deletion of TET1 in two independent TNBC cell lines resulted in reduced expression of PI3K pathway genes, upregulation of immune response genes, and substantially reduced cellular proliferation, suggesting the dependence of oncogenic pathways on TET1 overexpression. It also uncovered TET1 as a potential oncogene that contributes to aberrant hypomethylation in cancer and suggests that TET1 could serve as a potential drug target for therapeutic intervention (78). Notch3 can act as a tumor suppressor in the BC epithelial cells. Recent work showed that non-CpG methylation as a crucial cause leading to notch3 transcriptional repression in TNBC using *in vitro* methylation combined luciferase activity assays (80). Anticancer drugs targeting other components of the epigenome, including histone deacetylation and histone methylation, have also been approved by the FDA, and many others are going through clinical trials (81).

#### 3.1.1 DNMT inhibitor-based epigenetic therapy for TNBC

DNMT inhibitor-based epigenetic therapy has gained significant attention in the realm of TNBC treatment, shedding light on its potential as a therapeutic strategy. DNMT inhibitors, such as azacitidine and decitabine, exert their effects by targeting DNMTs which are responsible for adding methyl groups to cytosine residues in DNA leading to altered gene expression patterns. DNMT inhibitors offer a promising approach to reverse these epigenetic alterations and restore the expression of silenced genes, ultimately inhibiting tumor growth and sensitizing TNBC cells to conventional chemotherapeutic agents and targeted therapies. Recent studies have provided compelling evidence supporting the efficacy of DNMT inhibitor-based epigenetic therapy in preclinical models of TNBC. For instance, research conducted by Muvarak et al., demonstrated that treatment with decitabine restored BRCA1 expression and sensitized TNBC cells to PARP inhibitors, resulting in enhanced DNA damage and apoptosis (82). Similarly, Singh et al., reported that combination therapy with azacitidine and chemotherapy resulted in synergistic antitumor effects in TNBC xenograft models, highlighting the potential of DNMT inhibitors as adjunctive treatments for TNBC (83).

Moreover, clinical trials investigating the efficacy of DNMT inhibitor-based therapies in TNBC patients have shown promising results. A recent phase II clinical trial conducted by Luke et al., evaluated the safety and efficacy of azacitidine in combination with standard chemotherapy in patients with advanced TNBC (84). The study reported encouraging response rates and prolonged progression-free survival in patients receiving the combination therapy, underscoring the potential clinical benefit of DNMT inhibitor-based epigenetic therapy in TNBC. Despite these promising findings, challenges remain in the implementation of DNMT inhibitor-based epigenetic therapy for TNBC. One major hurdle is the identification of predictive biomarkers to stratify patients who are most likely to benefit from treatment. Additionally, optimizing treatment regimens and minimizing off-target effects are critical considerations for enhancing the therapeutic efficacy and safety of DNMT inhibitors in TNBC. Withaferin A (WA), another DNMT inhibitor, is a plant-derived steroidal lactone that holds promise as a therapeutic agent for treatment of BC (85). DNA hypermethylation of corresponding CpG sites in certain tumor-promoting genes such as urokinase-type plasminogen activator (PLAU), ADAM metallopeptidase domain 8 (ADAM8), tumor necrosis factor (ligand) superfamily member 12 (TNSF12), and genes related to detoxification; glutathione S-transferase mu 1 (GSTM1) and mitochondrial metabolism malic enzyme 3 (ME3) genes correlate with receptor tyrosine-protein kinase ERBB-2 amplification (HER2)/estrogen receptor (ER)/PR status in primary BC tumors (85). Moreover, upon comparing differentially methylated breast cancer cell lines for WA responsive target genes with DNA methylation changes in different clinical subtypes of BC patients in the cancer genome atlas (TCGA), it was found that WA silences HER2/PR/ER dependent gene expression programs in primary BC. This silencing suppresses the aggressive TNBC characteristics with an improved therapeutic sensitivity (85). Previous animal studies showed that WA effectively inhibits tumor growth and metastasis across various cancer types at doses of 1–20 mg/kg, with synergistic effects when combined with chemotherapies, and is well-tolerated *in vivo* (86, 87). Clinical trials have also demonstrated WA’s safety and tolerability, including a Phase I trial in osteosarcoma patients at doses up to 216 mg. Additionally, a clinical study is assessing WA in combination with liposomal doxorubicin for recurrent cancers, evaluating its feasibility, tolerance, and treatment response (88). Thus, DNMT inhibitor-based epigenetic therapy holds promise as a novel treatment approach for TNBC offering the potential to reverse aberrant DNA methylation patterns and restore tumor suppressor gene expression. Recent preclinical and clinical studies have provided compelling evidence supporting the efficacy of DNMT inhibitors in TNBC, paving the way for further investigation and optimization of these therapies in the clinical setting.

### 3.2 Histone modification and TNBC

Histone modifications are covalent post-translational alterations that occur in the sites of histone proteins and are considered as one of the main epigenetic mechanisms in a wide spectrum of disease regulation (89). This is because these modifications influence gene transcription, chromatin remodeling and nuclear architecture. Histones are known as DNA-packaging proteins because of their functional role in the formation of nucleosomes, the structural units of chromatin. The chromatin structure provides a precise compact structure for the genome organization, and thus influences genes to be either activated or silenced. In addition, chromatin is not static and thus liable for changes in the confirmation up on histone modification. The histone proteins form an octamer of four core histone proteins (H2A, H2B, H3 and H4) in order to wrap around a 147-bp stretch of DNA (90). Histone proteins consist of a globular C-terminal domain and an unstructured N-terminal tail which are densely populated with basic lysine and arginine residues (91). The N-terminal tails of histone extend outward from the nucleosomal core and provide sites for post translational covalent modifications such as methylation, acetylation, ubiquitination, sumoylation, phosphorylation, citrullination, AD-ribosylation, deamination, formylation, and butyrylation to take place on several specific residues. The state of chromatin structure is affected by histone modifications due to the alteration of charge density between histones and DNA (92). Histone modification can result either in activation or repression of gene expression depending on the type of modification and the specific residue of the modification. For instance, transcriptional activation is reported in the acetylation of lysine residue (93). Histone modifications have an enzymatic regulation where specific enzymes add or remove covalent modifications to histone proteins. For example, Histone acetyltransferases (HATs) and Histone methyltransferases (HMTs) add acetyl and methyl groups respectively whereas histone deacetylases (HDACs) and histone demethylases (HDMs) remove acetyl and methyl groups accordingly. These histone modifying enzymes have a specific connection with each other as well as other DNA regulatory mechanisms to ensure a tightly linked chromatin and gene expression status, chromatin organization and cellular identity (92, 94).

Moreover, histone acetylation is a major molecular epigenetic mechanism which affects gene expression through its effect on chromatin conformation. The ϵ-amino groups of lysine residues (e.g.: H2A; lysine 5,12,15 and 29 of histone H2B; 9,14,18 and 23 of histone H3 and lysine 5,8,12,16 and 20 of histone H4) (95, 96) located in the N-terminal extensions of core histone proteins provide sites for acetylation. The acetylation status of the targeted lysine is regulated by two counteracting enzymes: HAT and HDAC (92, 96). In addition to the enzymatic regulation of histone acetylation, these respective enzymes also regulate the acetylation of non-histone proteins (e.g., p53, Rb) (92). The ϵ-amino groups of lysine residues in histones H3 and H4 are subjected to the acetylation process in the presence of HAT which requires acetyl-CoA as a coenzyme to catalyze the enzymatic addition of acetyl groups. In addition, HATs play a crucial role for the acetylation of histone and non-histone proteins. HATs consist of five different families of acetylases namely, the p300/CBP family, the MYST family, the SRC family, the TAFII250 family, and GNAT family members. Each subfamily of HAT consists of transcription factors and steroid receptor coactivators with catalytic activity. However, the MYST family, the GNAT family and p300/CBP family are only found in human cells from all five subfamilies of HATs (97). There are three distinct types of chromatin configurations associated with histone acetylation and gene expression: active, repress, and bivalent. The active state of chromatin occurs via histone acetylation and resembles the open chromatin structures which is linked with active gene transcription, whereas the repressed state of chromatin results via histone deacetylation and represents the closed chromatin configuration which is associated with suppression of gene transcription. The bivalent state of chromatin consists of repressive and active histone markers which is predominant in developmental genes.

When considering the role of histone acetylation for active gene transcription, acetyl groups (COCH3) are transferred to lysine on N-terminal tails of histone by the activity of HATs. In addition, histone acetylation stabilizes the binding of chromatin remodeling factors at promoter regions and induces nucleosome unfolding as well as reduces nucleosome occupancy. The N-terminal side chains of histone core proteins are positively charged and thus readily interact with negatively charged genomic DNA. Ultimately, the positive charge on histones decreases with the acetylation and drastically reduces the interaction between genomic DNA and histones, resulting in the structural modification of the nucleosome which resembles the relaxed conformation of chromatin (euchromatin). Histone acetylation is abundant in the promoter regions of active genes and effects two major steps of gene transcription: initiation and elongation. The functional antagonists of HATs, HDACs remove the acetyl groups (histone deacetylation) restoring the interactions between genomic DNA and histones, resulting in a compressed chromatin structure (heterochromatin) and subsequently leading to the suppression of gene transcription (94). There are four main families of HDACs termed as class I, II, III, and IV. Class I, II, and IV are Zn^2+^ dependent whereas class III/Sirtuins are nicotinamide adenine dinucleotide (NAD)-dependent (98).

#### 3.2.1 Histone modification and cancer

Histone modification is an important epigenetic mechanism which plays a crucial role in the development and maintenance of tissue specific gene expression patterns in mammals. The aberrant changes in histone modifications directly cause the gene alterations and malignant cellular transformation (92). Histone modification is considered a vital epigenetic modification in cancer studies as the alterations in the patterns of histone post-translational modifications have been substantially linked to cancer. This is due to the fact that acetylation of histones largely occurs at the sites of enhancers, promoters, and gene bodies, thus alterations in global levels of histone acetylation result in aberrant gene expression (91). Based on evidence, histone acetylation at the sites of H4, lysine (K)16 is extensively linked to cancer phenotype in different types of cancers (99). Histone hyperacetylation affects the activation of proto-oncogenes whereas the histone hypoacetylation affects the silencing of TSGs that are localized to promoter (91).

The tight regulation of HATs and HDACs are vital processes for the prevention of cancer since the epigenetic driven tumor genes result in the alteration of enzymatic activities. The loss of regular functions of these enzymes results in the aberrant gene expression in eukaryotic cells, thus negatively altering normal cell functions such as cell cycle, differentiation, apoptosis, and proliferation. For instance, global loss of acetylation at K16 and trimethylation at K20 of histone H4 is considered a common abnormality in human cancer (99) and the reduced level of histone H3 lysine 18 acetylation (H3K18Ac) functions as a predictor of poor survival in pancreatic, breast, prostate and lung cancers (77). An altered acetylation results from three main circumstances based on the aberrant activities of HDACs and HATs: (i) abnormal recruitment of HDACs in the loci of TSGs (ii) reduced activity of HATs in the loci of TSGs, thus resulting the gene silence (iii) increased activity of HAT in the loci of oncogenes (100).

The mammalian HDACs perform important roles in gene transcriptional regulation, cell growth and survival. Thus, the aberrant expression of HDACs affects the balance of two major biological activities namely, enzymatic activity and functional activity. It has been found that the loss of the regular function of HDACs has been associated with cancer progression. In cancer cells, a global reduction in histone acetylation takes place due to the overexpression of HDAC whereas mutations are rare in HDACs. The role of HDACs in cancer prognosis consists of various types of tasks including the regulation of apoptosis in different types of cancer cells through changes in the expression levels of pro- and antiapoptotic proteins and reversibly modifying the acetylation status of histone and non-histone proteins, thus resulting in a broad range of aberrant gene expression patterns (101). In cancer cells, a global reduction in histone acetylation takes place due to the overexpression of HDAC.

In addition to the function of HATs as protein modifiers and epigenetic factors, they play a crucial role in multiple cellular processes, including proliferation, differentiation, growth arrest, apoptosis, and carcinogenesis (102). The most abundant HATs consist of p300 and CBP that involve in maintaining multiple cellular processes (103). Therefore, an anomalous expression of p300 and CBP is common in cancer cells where decrease of the expression during chemical hepatocellular carcinoma and mutations in p300/CBP occur at a considerable rate (104).

#### 3.2.2 Histone modifications and breast cancer

**Figure 3:**
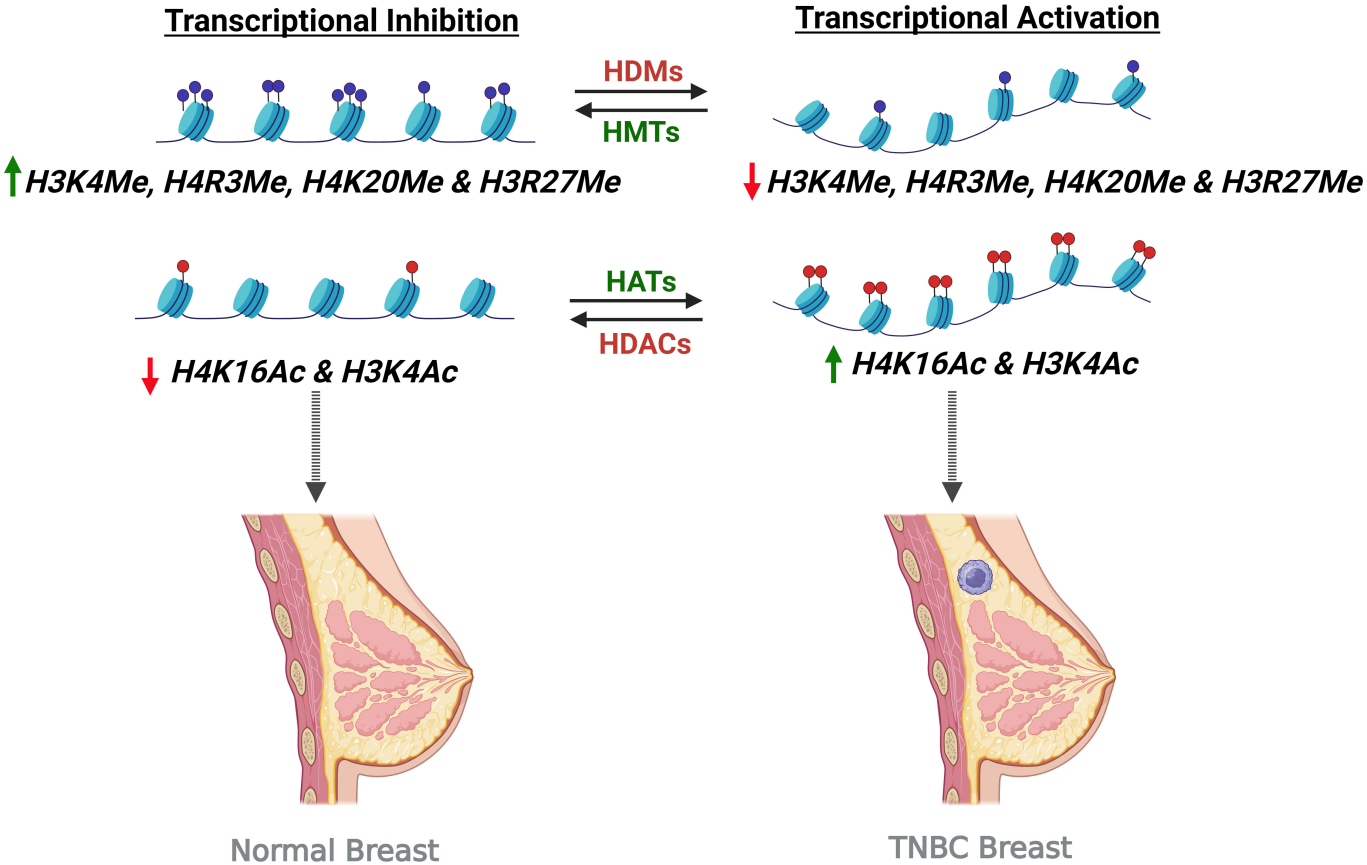
Histone modifications leading to transcriptional inhibition and activation in causing TNBC condition. Transcription activation upon increased histone acetylation by Histone Acetyltransferases (HATs) and decreased histone methylation by Histone Demethylases (HDMs) turns on the gene, leading to the TNBC condition. On the other hand, transcription inhibition by the activity of Histone Methyltransferases (HMTs) and Histone Deacetylases (HDACs) causing increased histone methylation and histone deacetylation respectively result in transcription inhibition and thereby preventing the TNBC formation.

Histone modifications and the aberrant histone acetylation directly affect the formation of BC. For instance, global reduction of monoacetylated lysine 16 of histone H4 (H4K16) and low levels of H4K16 acetylation are two major occurrences in early-stage BC (**Figure 3**). Moreover, H3K4 acetylation is identified in both early and later stages of BC phenotypes. When considering the role of histone acetylation for BC prognosis, a close relationship can be found in the development and treatment of BC. Histone acetylation is an important epigenetic mechanism that is responsible for the activation of gene expression (105). This is due to the formation of an euchromatin that accesses transcriptional factors to promoter regions and activates gene expression. Anomalous activation of certain genes via histone acetylation strongly supports the formation of BC. In particular, HATs can function as tumor suppressors, allowing cells to control proliferation and cell cycle and also as oncogene activating malignant proteins via aberrant histone acetylation (100). For instance, one of the families of HATs (p300) causes the activation of several oncogenes in human BCs. Thus, an intense expression of p300 can be observed in primary BCs (106). On the other hand, histone acetylation causes the inhibition of BCs. This can be understood by the fact that HATs of the family p300 result in an increase in the expression levels of Catechol-O-methyltransferase (COMT) gene. COMT protein is a vital enzyme which catalyzes the conversion and increases the metabolic rate of estrogen. Therefore, the histone acetylation which is catalyzed by p300 HAT causes a decline in the proliferation of MCF-7 BC cells that are stimulated by estrogen (107).

Furthermore, HDACs are responsible for some alterations in chromatin structure, gene expression regulation, and the proliferation of BC (108). The aberrant function of HDACs can promote BC formation by activating the expression of autophagy related genes (e.g.: *GABARAPL1*gene). Apart from HDAC’s role as a BC promoter, it has the potential to function as an inhibitor for the occurrence of BC. For instance, the vascular endothelial growth factor (VEGF) is an important factor that promotes angiogenesis in some pathological conditions including cancer. It has been found that transcription factor KLF-4 has access to recruit HDAC2 and HDAC3 at the VEGF promoter, promoting a decline in the expression of the VEGF gene then, resulting in an inhibition of BC prognosis (109). In addition, HDACs play an important role repressing metastasis associated gene expression, thus suppressing BC progression and metastasis (107). Additionally, EZH2, which is a transcriptional repressor and a histone methyltransferase, plays a role in cell cycle regulation and is associated with aggressive breast cancer. When compared to other non-TNBCs, elevated levels of EZH2 are strongly linked to the TNBC phenotype and tumorigenesis while the lower expression leads to poor patient survival. EZH2 catalytic inhibitors, such as Tazemetostat and GSK126, target the methylation activity of EZH2 but have minimal impact on its tumorigenic functions. As a result, they show limited effectiveness against most solid tumors and are particularly inadequate in inhibiting the growth of TNBC cells that rely on EZH2 (110, 111). However, it remains unclear whether specific genetic variants of EZH2 are associated with breast cancer risk.

#### 3.2.3 Therapeutic aspects

**Figure 4:**
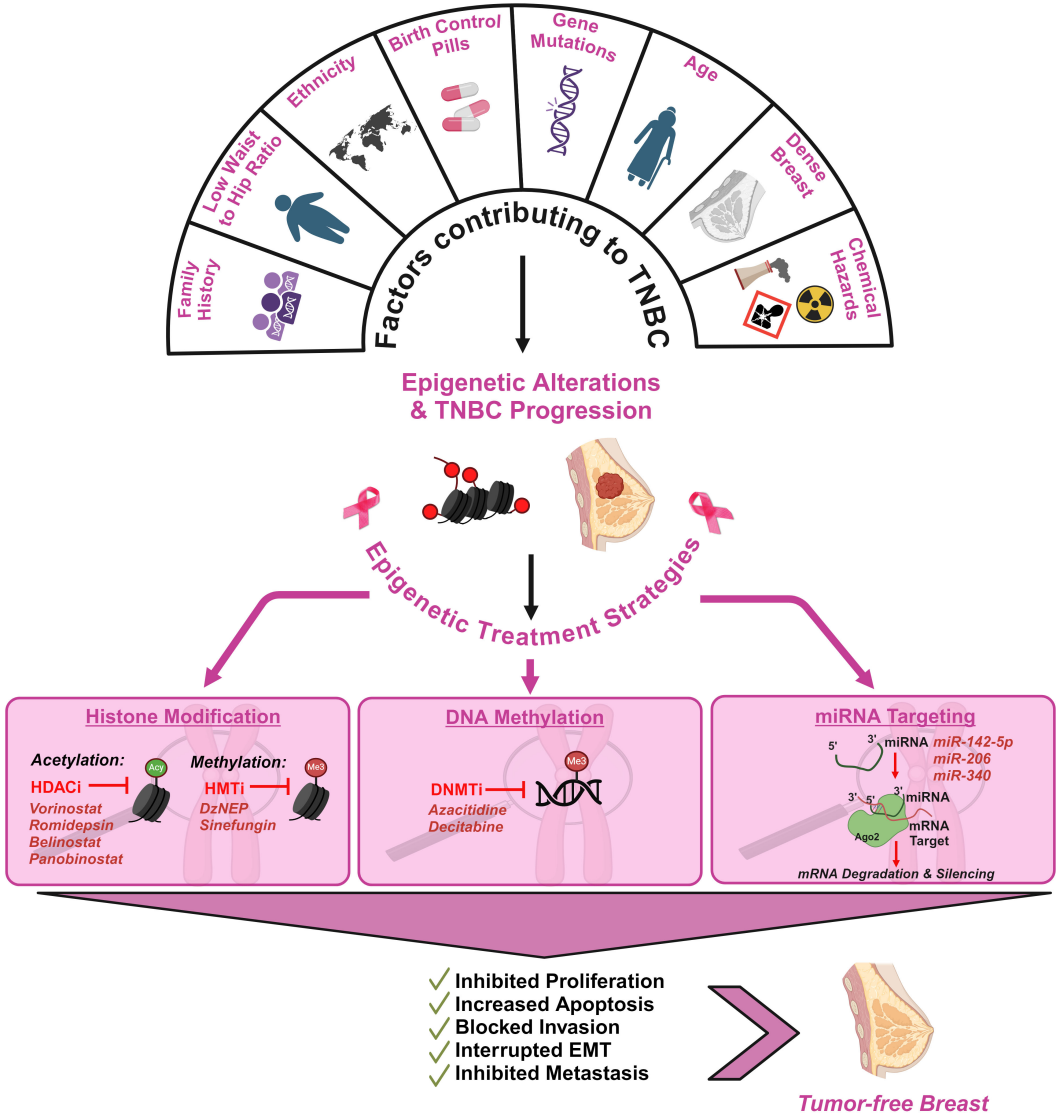
Schematic illustration of the factors contributing to TNBC and the impact of epigenetics in therapeutic interventions of TNBC. TNBC development is influenced by multiple genetic and epigenetic factors. Family history of breast cancer, low waist-to-hip ratio in women, use of birth control pills over 10 years, density of the breast and low socio-economic status have been highlighted as critical factors (113, 114). Epigenetic alterations associated with TNBC can be strategically targeted to reverse its progression. Histone Deacetylase Inhibitors (HDACi), Histone Methyl Transferase Inhibitors (HMTi), DNA Methyltrasferase Inhibitors (DNMTi) and microRNAs (miRNAs) are promising epigenetic therapies in mitigating TNBC tumor development (115, 116).

Histone deacetylation plays a major role in cancer prognosis and is often considered an ideal anti-cancer target (112). In addition, histone deacetylase inhibitors (HDACi) can interact in the catalytic domain of histone deacetylases thus resulting in a significant change in the acetylating activity of HDACs and the acetylating activity of HATs. The HDACis have an ability to impede the aberrant acetylation status of proteins that are found in cancer cells and restore the expression of tumor suppressors, apoptosis, differentiation and inhibition of angiogenesis and metastasis (112) (**Figure 4**). One of the main reasons for utilizing HDACis as therapeutic agents of cancer is that the sensitivity of cancer cells towards HDACi-induced apoptosis compared with normal cells. The treatment of tumor cells via HDACis exhibit two main pathways namely, the direct activation of apoptosis through extrinsic (death receptor) and intrinsic (mitochondria) pathways, and the second main pathway is the enhancement of the susceptibility of tumor cells to apoptosis (117, 118). However, HDACis do not possess an ability to act on the specific target; instead, they can target different pathways in cancer cells. For instance, the intrinsic and extrinsic pathways in the apoptosis process can also be affected by the HDACis and result in apoptosis induction via the upregulation of apoptotic proteins and downregulation of antiapoptotic proteins. The HDACis have access to mediate the major cellular functions including growth, differentiation, and survival. They can also be categorized into hydroxamic acids, cyclic tetrapeptides, benzamides, and electrophilic ketones based on their chemical structures.

The mechanism of HDACis in TNBC patients has not yet been classified. Yet, the mechanism is basically targeted towards chromatin remodeling through deacetylation of histones that determine the compaction of chromatin which is responsible for the prevention of transcription (119). In addition, the HDACis can target DNA and induce DNA damage, exhibiting a mechanism of oxidative stress or downregulation of proteins that are involved in the repair of oxidative damage (120). The wide role of HDACis also consists of the property of interfering with the intrinsic and extrinsic pathways of apoptosis and promoting apoptosis induction through upregulation of proapoptotic proteins and downregulation of antiapoptotic proteins. Moreover, HDACis have an anti-angiogenic effect thus decreasing the expression of VEGF receptors and inhibiting proliferation, invasion, migration, and adhesion of endothelial cells (121).

Histone modifications play an important role as an ideal therapeutic agent to treat patients diagnosed with TNBC. This is due to some of the important features in TNBC patients which create troublesome conditions for treating TNBC. For instance, these patients do not manifest positive responses to either endocrine therapy (tamoxifen and aromatase inhibitors) or targeted therapies (Trastuzumab and Lapatinib) due to the lack of presence in therapeutic agents. The major reason which lies behind the lack of responses to the above therapies is the presence of mutations in the tumor suppressor p53 (TP53) gene thus leading to the overexpression of mtp53 protein in TNBC cells. This mutation is reported approximately about 62% of the basal-like TNBC and 43% of the non-basal like TNBC (122). The role of mutation in p53 is that it readily involves resistance to apoptosis and inhibition of autophagy, thus manifesting gain-of-function mutation in p53 and resulting mild responses to typical therapies used in cancer treatments. Therefore, the down regulation of mtp53 remains the main strategy when considering potential therapeutic agents. In that sense, histone deacetylase inhibitors play A recent study revealed that HDACis inhibit the proliferation of TNBC cells through cell cycle arrest and mitochondria-related apoptosis. Among the various HDACis, Suberanilohydroxamic acid (SAHA) (94) and NaB [Nanoparticle albumin-bound paclitaxel (nab-paclitaxel)] (123) are the most widely used anticancer agents for treating TNBC patients. This is based on the findings on the use of SAHA and NaB to suppress the proliferation, arrest cell cycle progression, and induce mitochondrial related apoptosis in TNBC cells as well as their capabilities to decrease the phosphorylation, protein, and mRNA levels of mtp53. In addition, the downregulation of mtp53 transcription is also mediated by the acetylation of YY1 at residues 170-200 by HDAC8 (122). Therefore, the use of SAHA and NaB is an ideal application as both of these inhibitors have access to disrupt the interaction between HDAC8 and YY1, thus increasing the acetylation of residues 170-200 of YY1 and suppressing the YY1 induced p53 transcription (94, 122). Moreover, studies show that the combination of ionizing radiation (IR) and SAHA significantly enhances the therapeutic efficacy and increased DNA damage by DNA repair protein inhibition compared to treatment alone (124). Additionally, SAHA also inhibits lung metastasis in BC, suggesting that SAHA, either alone or in combination with IR, could be a promising therapeutic strategy for breast cancer. Further, a retrospective study conducted on HER2-negative BC patients underwent nab-paclitaxel treatment exhibited enhanced pathological complete response (pCR), particularly for patients with TNBC or lymph node-negative breast cancer (125). As NaB does not rely on non-ionic surfactants to solubilize paclitaxel, which are known to contribute to toxicity and trap paclitaxel within solvent-based micelles (126), NaB is considered safe and less toxic as a treatment option for TNBC patients.

In the light of recent research findings, the HDACis have shown limited responses when used as single agents, whereas the combination of kinase inhibitors, autophagy inhibitors, and ionizing radiation or the use of two different inhibitors is being evaluated by scientists (127, 128). For instance, entinostat (formally MS-275, Syndax Pharmaceuticals, Inc, Waltham, MA) is an ideal example of class I HDAC inhibitor which possesses the ability to reduce the proliferation of cancer cells and tumor xenografts in various cancer types while exhibiting mild toxicity to normal cells (129). However, there was confusion about entinostat’s ability to induce the expression of pro-apoptotic BIM protein in TNBC patients whereas a recent preclinical study has demonstrated the entinostat-induced expression of the pro-apoptotic BIM proteins in TNBC patients (130). In addition, an elevated level of NOXA (a member of the Bcl-2 family of apoptosis-regulating proteins) has been reported in about 65% of TNBC patients as response to the entinostat treatment (131). In cancer cells, the NOXA protein is in an epigenetically silent state due to the aberrant histone acetylation in cancer cells. Thus, it indicates a positive impact of histone deacetylase inhibitors in TNBC patients. Promising results have been reported from the ENCORE 301 randomized Phase II study (132), which evaluated the combination of entinostat and endocrine therapy in advanced BC. The study demonstrated a significant improvement in progression-free survival in the entinostat group and very well tolerated with reduced risk in patient groups (133). In addition, the mitogen activated protein kinase (MAPK) is activated in TNBC cells while the extracellular signal related kinase (ERK) is a member of the MAPK pathway which promotes cell proliferation, angiogenesis, differentiation, and cell survival (134). Therefore, the ERK pathway is known to be an important therapeutic target in TNBC. Moreover, a combination therapy of pimasertib (ERK inhibitor) and entinostat (HDAC inhibitor) is considered as promising therapy that reduces tumorigenic potential and proliferation (135).

The inherent heterogeneity of TNBC is well-documented at clinical, histopathological, and molecular levels. This genomic diversity, which includes variations in copy number changes, mutations, and chromosomal rearrangements, complicates the development of effective therapies (136). However, recognizing this heterogeneity is critical for improving treatment outcomes. Stratifying patients based on molecular and epigenetic profiles can help identify those most likely to benefit from specific therapies. As discussed earlier in this review, the different subtypes of TNBC, classified based on gene expression profiling, can respond differently to treatment. To tackle this challenge, it is vital to further investigate the tumor immune drivers specific to each TNBC subtype and stage (136). A deeper understanding of these immune factors will enable us to tailor immunotherapies more effectively for patients with TNBC in a more personalized manner. A study by Hu et al., explored the different subtypes of TNBC and found significant differences in tumor genetics, immune cell compositions, cytokine profiling, sensitivity to immunotherapy and chemotherapy, across these subtypes resulting in notable variations in clinical prognosis (137). These findings offer valuable insights for developing personalized therapeutic strategies and improving prognosis evaluation for TNBC patients in the future. Personalized treatment approaches, which consider the unique characteristics of a patient’s tumor, will offer success for overcoming resistance and maximizing the efficacy of epigenetic interventions. Thus, customizing treatments, based on the specific subtype and epigenetic landscape of TNBC, allows for more precise targeting of the underlying mechanisms driving tumor progression, ultimately improving long-term outcomes for patients.

Further, it is also important to keep in mind that cancer cells can also acquire resistance to epigenetic therapies, which can then interfere with the expression of genes involved in cell cycle regulation, DNA repair, and apoptosis, making therapies become inefficient. Resistance to epigenetic therapies in TNBC can arise through various mechanisms. Changes in epigenetic regulators, such as mutations or overexpression of HDACs, DNMTs, and other chromatin modifiers (138), can restore tumor suppressor gene silencing, reducing the effectiveness of treatment. Additionally, TNBC cells may activate compensatory signaling pathways, including PI3K/AKT and MAPK/ERK, and exhibit epigenetic plasticity, which allow them to adapt and evade therapy. The TME can become more immunosuppressive, with increased expression of immune checkpoints like PD-L1 and the recruitment of regulatory T cells and myeloid-derived suppressor cells (MDSCs) (139), further impairing therapeutic efficacy. Other contributing factors include the reversibility and redundancy of epigenetic changes, activation of DNA repair pathways, increased drug efflux, and altered metabolism. Additionally, the re-silencing of tumor suppressor genes and the induction of epithelial-to-mesenchymal transition (EMT) or cancer stem cell (CSC) properties can further promote resistance. These mechanisms underline the need for combination therapies to overcome resistance and enhance treatment outcomes in TNBC.

As pointed out earlier, paclitaxel used with epigenetic drugs such as SAHA (140); EAD (Entinostat, used in combination with ATRA (all-trans retinoic acid) and Doxorubicin) (141) (this combination treatment can restore epigenetically silenced RAR-β expression) are some examples of confirmed combined epigenetic therapies. Moreover, 5-azacitidine (5-AZA) with entinostat in combination therapy is still underway in phase II clinical trials (expected completion in 2025) (142). AZA and entinostat work through different mechanisms to inhibit tumor cell growth, either by directly killing the cells or by preventing their division. Entinostat may hinder tumor cell growth by blocking certain enzymes [class I and IV histone deacetylase (HDAC)] essential for cell proliferation. When used together, azacitidine and entinostat may enhance the destruction of tumor cells. In addition, epigenetic drug treatments along with immunotherapy are another approach to overcome this challenge. A combination of immune checkpoint inhibition and epigenetic therapy overcomes the limitations of immunotherapy and paves a way for the therapeutic resistance against epigenetic therapies (143). Epigenetic drugs, such as HDACi and DNMTi, have been shown to improve the effectiveness of immune checkpoint inhibitors. Epigenetic therapies can also increase the expression of immune checkpoint genes like PD-L1, making tumor cells more vulnerable to immune attack. Combining immune checkpoint inhibitors with epigenetic modifiers, such as EZH2 inhibitors and HDAC inhibitors, can amplify the immune response by enhancing antigen presentation, regulating immune cell function, and reducing immune evasion (144). Overall, these findings suggest that combining epigenetic therapies with immune checkpoint inhibitors holds significant potential for improving cancer treatment outcomes.

#### 3.2.4 Epigenetic drug delivery strategies in TNBC treatment

The delivery of epigenetic drugs in TNBC faces significant challenges due to the molecular heterogeneity, and the difficulty in achieving efficient drug delivery. Several novel strategies have been developed to improve the targeting, and therapeutic efficacy of epigenetic drugs to overcome these hurdles in the drug development (145). Nanoparticle-based delivery systems, such as liposomes (146), solid lipid nanoparticles (SLNs) (147), and polymeric nanoparticles (146), can encapsulate and protect epigenetic agents, improving their stability, solubility, and cellular uptake. This delivery method can increase drug delivery at the tumor site while minimizing the toxicity. Additionally, exosome delivery is being investigated as another novel method for maximizing targeted delivery, using the body’s biological machinery to enhance drug delivery in a more immune-tolerant manner (148). Exosomes are bio-vesicles composed of lipid bilayers that enable selective transport of therapeutic agents to tumor cells, improving treatment specificity and minimizing off-target effects. Their ability to cross biological barriers and the potential for surface modification for tumor targeting make exosomes an ideal platform for overcoming TNBC’s aggressive characteristics and drug resistance. Further, targeted drug delivery methods are more refined by conjugating epigenetic drugs to specific ligands, such as antibodies or peptides, that bind to overexpressed receptors on TNBC cells, improving the precision of drug delivery, and enhancing therapeutic outcomes (149). Responsive drug delivery systems, which release drugs in response to specific stimuli like pH changes or enzyme activity in the TME, are also some other promising approaches for TNBC drug delivery (150). Additionally, CRISPR-based gene editing technologies are being explored for their potential to modify the epigenetic landscape of TNBC cells directly, although safe and efficient delivery remain challenging (151, 152).

Other strategies, such as intratumoral injection, aim to bypass systemic circulation and deliver high concentrations of drugs directly to the tumor (153). Hence, integrating epigenetic modifications with treatments like radiation therapy, it may be possible to sensitize tumors to DNA damage and improve overall treatment effectiveness. These advanced delivery strategies aim to overcome the challenges associated with epigenetic drug administration, paving the way for more effective, personalized therapies for TNBC patients (154).

Although epigenetic therapies hold great promise in treating various diseases including TNBC, these therapies can have long-term effects that require careful monitoring of patients for possible adverse reactions and potential relapses (155). The dynamic nature of epigenetic modifications means that changes in gene expression may evolve over time, potentially leading to reversibility and re-establishment of abnormal patterns across the genome (156). Consequently, continuous medical assessment is essential to track alterations in patients’ epigenetic profiles and alleviate any risks. One of the high-risk factors is that epigenetic modifications are temporary and can revert to result in disease recurrence if not properly managed (157). While targeted drug delivery methods have shown promise in treating TNBC, off-target effects can lead to adverse side effects and complications that may become life-threatening (158). Moreover, changes to the epigenome using drug treatments might as well interfere with the normal cellular processes and could potentially result in secondary mutations with continued treatment for TNBC (159). Therefore, clinical trials in this respect need to justify the importance for assessing treatment efficacy, identifying potential resistance mechanisms, and detecting early indications of relapse.

### 3.3 Epigenetics and microRNAs: implications in cancer

miRNA is a class of endogenous non-coding RNA molecules of about 18-21 nucleotides in length that performs a regulatory role in most multicellular and complex eukaryotes including plants and animals (160). Typically, “mir” refers to precursor miRNA (pre-miRNA), while “miR” refers to the mature miRNA product. Since there can be multiple miRNAs which are evolutionary related, a letter after the number in the suffix is given to differentiate multiple members of the similar family. miRNA being a class of highly conserved molecule between species espouse that they have a universal role in regulating gene expression. The members of this group are negative regulators that control target gene expression through post-translational inhibition of RNA or degradation via imperfect complementarity to the 3’ UTR of the target mRNA (160). The presence of conserved 3’ UTR regions of mRNA molecules capable of binding to miRNA postulates possible miRNA-driven control of these genes (160, 161). While traditionally associated with the regulation of mRNA stability and translation, emerging evidence suggests that miRNAs also participate in epigenetic processes, influencing chromatin structure and gene expression patterns without altering the underlying DNA sequence. The primary involvement of miRNAs in epigenetic regulation revolves around modulating the function of proteins engaged in these processes. One mechanism through which miRNAs exert their influence is by targeting the mRNA transcripts of enzymes responsible for DNA methylation and histone modification. For instance, specific miRNAs can target DNMTs or HMTs, thereby regulating the addition of methyl or acetyl groups to DNA or histone proteins, respectively. Moreover, miRNAs indirectly affect epigenetic modifications by targeting transcription factors or other regulatory proteins that oversee the expression of epigenetic modifiers. Through the modulation of these pivotal regulators, miRNAs can shape the overall epigenetic landscape within cells or tissues.

Recent investigations have also revealed the involvement of miRNAs in controlling long non-coding RNAs (lncRNAs) and circular RNAs (circRNAs), which themselves serve as epigenetic regulators (162). miRNAs can interact with lncRNAs or circRNAs to establish regulatory networks that influence chromatin structure and gene expression. Significantly, the dysregulation of miRNA-mediated epigenetic regulation has been implicated in various diseases, including cancer, neurodegenerative disorders, and cardiovascular diseases (163, 164). Anomalous miRNA expression can disrupt normal epigenetic patterns, leading to altered gene expression profiles and contributing to the development of disease. To sum up, miRNAs exhibit a multifaceted role in epigenetic regulation by targeting key components involved in DNA methylation, histone modification, and chromatin remodelling pathways. A comprehensive understanding of the intricate interplay between miRNAs and epigenetic processes is essential for unravelling the molecular mechanisms underlying development, disease pathology, and the exploration of potential therapeutic avenues. miRNA mediated gene silencing may occur in one of three ways.

**Figure 5:**
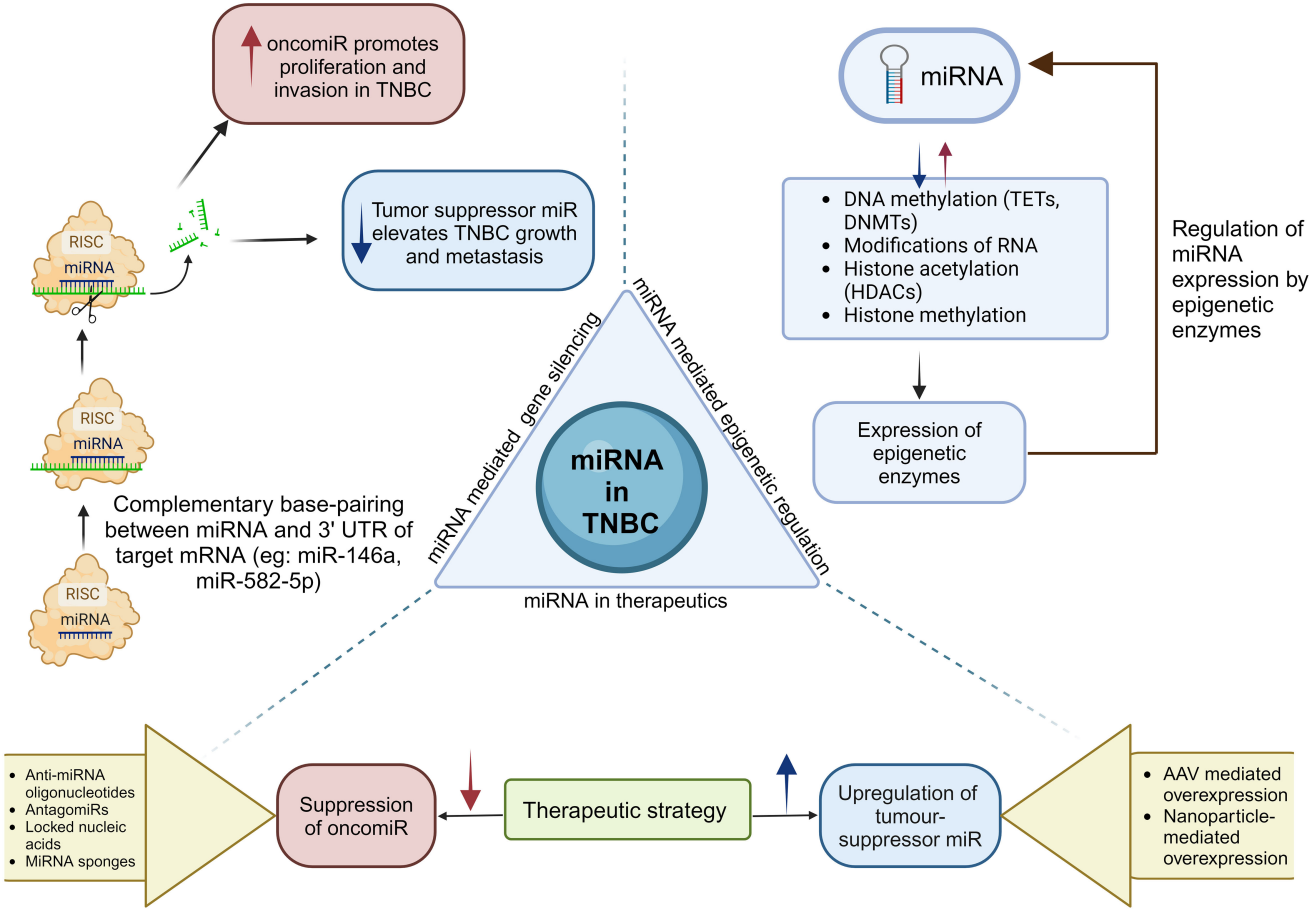
Biogenesis of miRNAs leads to the formation of oncomiRs and tumor suppressor miRs. miRNAs play a crucial role in epigenetics by modulating epigenetic enzymes, which subsequently influence the expression levels of miRNAs. The critical role of miRNAs in TNBC highlights the requirement for miRNA-based therapeutic strategies that increase tumor suppressor miRNAs or reduce oncomiRs (171, 172).

Involvement of miRNA in cancer development was first reported in 2002 with the finding that the miR-15a and miR-16-1 are lost in the B cells of 70% of chronic lymphocytic leukemia patients due to the translocation induced deletion of chromosome 313q14.3 resulting in an increase in anti-apoptotic gene BCL2 product (165). Subsequent research has classified miRNAs into two distinct categories: oncogenic miRNA (oncomiR) and tumor- suppressive microRNAs (tumor suppressor miR) (166–168). OncomiRs are genes that are usually upregulated in most cancer types, leading to TSG product degradation while promoting cancerous phenotypes including cell proliferation, invasion, resistance, and apoptosis (169). Tumor suppressor miRs are genes involved in anti- tumor function via targeting oncogenes to degradation, that get downregulated during cancer (169, 170) (**Figure 5**). Genome-wide miRNA expression profiles have shown deregulated expression of miRNA in all cancer type studied so far, implying their function as either tumor suppressor miRs or oncomiRs (173). Localization of >50% of the annotated human genes in the fragile chromosomal sectors make them susceptible to deletion, translocation, and amplification during tumor progression (174). Research aimed at identifying miRNA expression profiles in various tumor types has provided new insights into the distinct expression patterns of miRNA associated with the differentiation states and developmental lineage of tumors (175). In addition, the downregulation of miRNAs in most cancer types, tumor promotion with knockdown of miRNA processing components both *in vitro* and *in vivo*, and poor prognosis with reduced expression of Dicer in certain lung cancer imply the necessity of identifying novel miRNAs whose functionality is associated with cancer suppression (170, 176, 177). Furthermore, the ability to classify poorly differentiated tumor types using the miRNA expression profile in contrast to the mRNA profile, being small and stable, and the ability to be obtained from formalin fixed paraffin embedded samples, frozen samples, as well as blood, makes miRNA a novel and promising candidate as a prognostic marker and potential drug target (178, 179).

#### 3.3.1 miRNA expression profiling techniques

The reported functionality of miRNA as biomarkers of prognosis and metastasis necessitates innovation of techniques for the quantitative and qualitative analysis of miRNA in cells. Two prevailing techniques for the identification of aberrant miRNA expression include expression profile study and functional screening assay (170). The basal point of expression analysis depends on the differential expression of the miRNA in cancerous tissues in comparison with non-cancerous counterparts and the specificity of miRNA expression profile in different cancer types (120–124). Expression analysis techniques include cloning, northern blotting, microarray, sequence- by- synthesis, serial analysis of gene expression (SAGE), *in situ* hybridization, and quantitative RT-PCR (qRT- PCR). Among these techniques, microarray is considered to be the most widely used experimental platform for miRNA expression profiling, while qRT- PCR is used for quantification of specific miRNA and genome wide miRNA (170). The length of mature miRNA is short (~22 nt) which makes discrimination between individual members of a miRNA family difficult. The lack of a standardized normalization method in microarray techniques, along with the poor correlation between altered miRNA expression profiles and their functionality in cancer as shown in functional assays, necessitates a more precise method to link miRNA with cancer prognosis (180, 181). In contrast, functional screening assays that rely on identifying ‘driver’ miRNA linked to the specific phenotype of interest independent of expression level, make them a promising candidate for identifying targets in novel cancer therapy. Functional screening assay involves the introduction of miRNA into the cells and determination of cancer related phenotypes to respective miRNAs (170). Integration of both approaches can lead to successful future studies focusing on cancer and the development of novel therapies and biomarkers.

#### 3.3.2 miRNA profiles in TNBC progression

The first study to demonstrate the involvement of miRNA in the development of BC was carried out by Iorio and colleagues who identified altered miRNA expression in BC cells in comparison to non-cancerous cells and the correspondence of many miRNA expression profiles to BC subtypes and clinical pathological features (182). Since TNBC is an aggressive BC subtype, it is imperative to identify novel biomarkers for the early detection and studies concerning miRNA profiling with TNBC tumor progression have taken a new lead. Many studies have been carried out in identifying miRNA expression profiles, and the functional and prognostic characteristics in TNBC. These studies focus on identifying specific miRNAs that are differentially expressed in TNBC in comparison to other BC subtypes. To date, many miRNAs involved with the three primary missing receptors (ER, PR and HER2) of TNBC and cancer susceptibility gene BRCA1 have been identified and reported (178).

Several studies have been undertaken to identify the miRNAs’ expression profiles to distinguish TNBC from other BC subtypes. In this section we summarize the studies carried out to identify miRNAs in association with TNBC and three receptors (ER, PR, and HER2). A study carried out with the aim of identifying altered miRNA profiles in TNBC that vary in ER, PR, and HER2/neu receptors status. Expression profiling of 453 miRNAs from 29 early-stage BC cases resulted in the identification of the miRNA signatures associated with the ER status (miR-342, miR-299, miR-217, miR-190, miR-135b, miR-218), PR status (miR-520g, miR-377, miR-527-518a, miR-520f-520c) and HER2/neu (miR-520d, miR-181c, miR-320c, miR-376b, miR-30e). Further analysis of miR-342 and miR-520g in 95 breast tumors resulted in the identification that miR-342 expression is lowest in TNBC in comparison to ER and HER2/neu positive luminal breast tumors while miR-520g is up regulated in ER and PR negative tumors (183). Another study concerned with miRNA signature in 103 lymph node-negative BCs showed an association between the TNBC subtype miR532-5P, miR-500, miR362-5p, and miR502-3p which are located at Xp11.23 and members of miR-17 to be upregulated in ER-negative tumors (184). In addition, the findings also included miRNAs associated with different clinicopathological features reporting tumor size, Nottingham grades 1 through 3, mitotic activity index, positive vs negative for the TNBC, ER, PR, Her2/neu where 45 miRNAs are associated with TNBC (184).

The study of altered miRNA expression in the epithelial cell subpopulation of BC using 100 TNBC samples showed decreased accumulation of miR-145 and miR-205 (185). A study on identifying novel miRNAs associated with TNBC using integrated genomic analysis identified seven miRNAs linked with TNBC prognosis [miR-17-92 cluster (miR-20a, miR-92a, miR-17/*, miR-19a/b, miR-18a), miR-106b-25 (miR-106b, miR-93)] (186). Investigation of miRNA-93, 190, and 200b in relation to receptor status in stage III BC revealed up regulation of miRNA-93 in ER and PR negative patients (187). Analysis of 16 miRNAs in TNBC samples in the identification of lymph node metastasis reported miR-200c and miR-205 as potential biomarkers for lymph node metastasis determination in BC. This study further demonstrated that miR-34a, miR-34c, miR-181a and miR-146a were downregulated by 1.5-fold in TNBC while no changes in expression was significant either in metastatic or nonmetastatic tumors. Conversely, an upregulation in expression was observed in miR-146b and miR-122 in metastatic tumors although it was not significant enough. Furthermore, miR-155 which was reported to be upregulated from another study was known for metastases in BC patients (188). miR-21 was overexpressed by 1.8-fold in tumor tissues although the increase in expression in metastatic and nonmetastatic tumors was not significant. Additionally, miR-9, miR-100, miR-30a and miR-30d didn’t illustrate a significant change in expression in tumors. Hence, a 1.5-fold down regulation of miR-31, miR-205, miR-34a, miR-146a, miR-125b, miR-34c, and miR-181a and 1.5-fold increased accumulation of miR-21 were found in TNBC tissues in comparison to benign breast tissue (189).

Remarkably, a study examining tumor suppressor miRNAs implicated in lymph node metastasis in TNBC conducted comparisons among 31 primary TNBC cases, 13 lymph node metastasis samples and 23 normal breast tissues (190). Results depicted dysregulation of 71 miRNAs in which the majority were already reported to be involved in BC development, suggesting non-subtype specific miRNA involvement in TNBC development. In addition, the differential expression of 27 miRNAs in lymph node metastasis with 25 of them being downregulated was also reported (190). TNBC specific integrated miRNA and mRNA signature identification studies led to the finding of 116 miRNAs deregulated in TNBC with upregulation of miR-106b, miR-17/92 cluster, miR-200 family, miR-21 and miR-155 and downregulation of let-7b, let- 7c, miR-126, miR-145 and miR-205 (**Table 1**). Furthermore, miR-424, miR-125a-5p, miR-627, miR-579, let-7g and miR-101 were found to be involved in metastasis (204). A study involving meta-analysis approach has identified 6 dysregulated miRNAs in which 4 miRNAs (hsa-miR-135b-5p, hsa-miR-18a-5p, hsa-miR-9-5p and hsa-miR-522-3p) are upregulated and 2 miRNAs (hsa-miR-190b and hsa-miR-449a) are downregulated (205). In a study involving serum level analysis of miRNAs (miRNA-21, miRNA-10b, and miRNA-200c) has shown that the miRNA-200c is downregulated in TNBC in comparison to ER-positive/PR-positive control group. No significant differences were observed in the other two miRNAs analyzed (miRNA-21 and miRNA-10b) (206). Analysis of the expression level of 19 miRNAs in cancer versus normal breast tissues have shown downregulation of miR-190a, miR- 136-5p, and miR-126-5p and upregulation of miR-135b-5p and miR-182-5p among the analyzed miRNAs. Furthermore, a correlation has been observed between the expression levels of miR-126-5p and miR-135b-5p and tumor sizes.

**Table 1:** TNBC related functional studies on tumor suppressor miRNAs.

miRNA	Outcome	Reference
miR-200a/miR200b	Downregulation of transcriptional repressors (zinc-finger E-box–binding homeobox 1) *Zeb1, Zeb2* and *Suz12*) of *E-cadherin* that inhibit epithelial-mesenchymal transition (EMT). Downregulation of *EphA2* mRNA- mammary gland branching, mammary tumorigenesis, and metastasis. miR-200b involves Cα mediated suppression of metastasis in TNBC.	(191, 192)
miR-200c	Downregulation of *Zeb1, Zeb2, FN1, MSN, TrkB.* This inhibits EMT and invasion.	(193, 194)
miR-205	Downregulation of E2F1 (involve in the phase change from G1 to S phase in the cell cycle), LAMC1 (cell adhesion, differentiation, migration, proliferation, signaling, neurite growth, metastasis).	(195)
miR-203	Suppression of proliferation, mobility of TNBC cells by downregulating BIRC5 and LASP1.	(196)
miR-31	Inhibition of cell migration, invasion and metastasis by inhibiting oncogenic NF-ĸB pathway via down-regulating protein kinase C epsilon (PKCε).	(140)
miR-34a	Interaction with AXL mRNA inhibits cell migration.	(197)
miR-200 family	Downregulation of activators of EMT, transforming growth factor β2 and ZEB1. Promotes cancer cell invasion.	(198)
Let-7 family	Regulation of cell proliferation and differentiation. Targets oncogenes *RAS*, *HMGA2* and MYC.	(199)
miR-200b	Inhibition of cell migration and metastasis by the downregulation of protein kinase Cα.	(191)
miR-200c	Targets X- linked inhibitor of apoptosis (XIAP). Involve in cell proliferation and induction of apoptosis. Downregulated in tumor tissues.	(200)
miR-22-3p	Downregulated in TNBC. Restoration of the expression results in inhibition of TNBC cell proliferation, colony formation, motility, invasion and phosphatidylinositol 3-kinase/Akt and Src signaling. miR-22-3p inhibits the expression of eukaryotic elongation factor 2 kinase (eEF2K).	(201)
miR-192	Downregulated in TNBC. Overexpression prevents proliferation and migration and promotes cell apoptosis. Found to bind with Rho GTPase Activating Protein ARHGAP19 which is found to prevent migration upon downregulation.	(196)
miR-33a	Overexpression in TNBC cells significantly prevents cell growth, migration and promote cell cycle arrest in G1 phase.	(202)
miR-590-3p	Downregulated in TNBC. Cell migration, invasion, metastasis is inhibited when overexpressed. Targets 3’UTR of Slug which is a key player of EMT.	(203)

#### 3.3.3 miRNAs as predictive and prognostic biomarkers for TNBC

miRNAs serve a dual role as predictive and prognostic biomarkers in TNBC offering invaluable insights into disease progression and patient outcome. TNBC-based studies have shown that increased levels miR-16, miR-155, and miR-374 can act as positive prognostic biomarkers for TNBC patients. At the same time, miR-125b is a prognostic marker where its decrease indicates poor prognosis. Subsequent classification has resulted in the grouping of prognostic biomarkers miR-125b, miR-655, and miR-421 as a risk associated miRs and miR-16, miR-37a, b, and miR-497 as protective miRs (204) (**Table 2**). The negative correlation of disease-free survival (DFS) and OS with miR-34b presents miR-34b as a promising prognostic biomarker in TNBC (217). The loss of E-cadherin functionality by ZEB1 (transcription factor controlling the differentiation of cancer cells, and metastasis) (217) and ZEB2 (a role in epithelial-mesenchymal transition–dependent tumor metastasis) (218) during epithelial-to-mesenchymal transition (EMT), leads to enhanced migration and invasion, influencing cancer prognosis. This effect, attributed to decreased levels of the miR-200 family, classifies them as predictive biomarkers (219). TNBC tissue-specific studies have identified miR-342, miR-150 and miR-27b as good prognostic biomarkers (**Table 2**) and miR210, and miR-144 as poor prognosis biomarkers (220). Research has identified higher expression of miR-200b-3p, miR-190a and lower expression of miR-512-5p in combination as a better chemotherapy response with increased feasibility of breast-conserving surgery (187). In TNBC, upregulation of miR-558 and downregulation of 4 miRNAs which include miR-21, miR-99a, miR-548v, and miR-320d-1 play a role in TNBC pathogenesis and are identified as potential biomarkers of the disease (221, 222). In a study analyzing differentially expressed miRNAs in TNBC vs matched normal tissues revealed 194 differentially expressed miRNAs, in which 3 miRNAs (miR−200b−5p, miR−21−3p, and miR−659−5p) were identified as potential biomarkers of TNBC (213) (**Table 1**). Research on identifying miRNAs correlating with relapse in TNBC patients after surgery identified a combination of 8 miRNAs (miR-139-5p, miR-10b-5p, miR-486-5p, miR-455-3p, miR-107, miR-146b-5p, miR-324-5p and miR-20a-5p) as potential prognostic markers (223). miR-223 has been identified as an independent prognostic marker-associated with improved OS and DFS in TNBC (224).

**Table 2:** TNBC related functional studies on oncogenic miRNAs.

miRNA	Outcome	Reference
miR181a/b	Promotion of aggressiveness of BC by inhibiting DNA damage response through deregulating stress sensor ATM.	(207)
miR-146a, miR146b-5p	Binds to 3’ UTR of *BRCA1* and down-regulates its expression leading to increased proliferation and reduced homologous recombination rates.	(208)
miR-182	Downregulation of PFN1 protein that involves cell proliferation, migration, cell viability and apoptosis.	(209, 210)
MiR-15a, b, miR-16, miR-128	Targets Smurf2 and inactivates retinoblastoma that involves in tumor suppression.	(211)
MiR-93	Targets tumor suppressors including large tumor suppressor homolog 2 (LATS2/KPM). Over expression correlates to poor prognosis. Ectopic over expression promotes proliferation, invasion, and migration.	(212)
miR−301a−3p	Upregulated in TNBC. Overexpression in MDA-MB-231 cells found to promote cell viability, migration and invasion and negatively regulate cell apoptosis. Shows negative correlation with the expression of mesenchyme homeobox 2 (MEOX2) which was found to promote cell viability upon knockdown.	(213)
miR-582-5p	Upregulated in TNBC. Overexpression leads to tumor growth and metastasis in *in vivo* models. Inhibits CMTM8 expression which has been shown inhibit tumor proliferation and invasion.	(214)
miR-27a-3p	Highly expressed on TNBC. *In vitro* analysis shows over expression leading to proliferation and migration. negatively regulates Wnt/β-catenin signaling pathway by targeting 3’ UTR of GSK3β mRNA.	(215)
miR-27b-3p	Elevated expression in TNBC. Promotes cell proliferation, migration, invasion, and metastasis. Found to promote EMT by activating Snail and NF-κB via suppressing peroxisome proliferator-activated receptor gamma (PPARG)	(216)

#### 3.3.4 miRNA based therapeutics

Involvement of miRNA in cancer initiation, development, and progression make them a potential candidate for drug development in miRNA-specific targeted therapies (178). MiRNA based therapeutic methods utilize either suppression of oncogenic miRNA or increasing tumor-suppressive miRNA levels. Oncogenic miRNA approaches based on the delivery of miRNA specific synthetic RNA analogues, result in the silencing of endogenous miRNA. These approaches include anti-miRNA oligonucleotides which are single-stranded molecules that inhibit target miRNA through direct complementarity (225). Another approach includes antagomirs which are antisense oligonucleotides chemically modified by conjugation with cholesterol conjugated 2’-O-methyl with increased stability. Antagomir based silencing are common in the silencing of miR-10b involved with metastasis (226). The locked nucleic acids are a new approach that utilizes oligomers with methylene bridges that functionally locks ribose conformation, increasing the stability and affinity of oligomers to a specific miRNA (225). The miRNA sponges are novel approaches in which a single sponge consists of multiple binding sites at the 3’ UTR of mRNA for a specific miRNA. Multiple binding sites allow control of multiple miRNAs, but factors such as vector size and poor distribution in the body restrict their usage in BC trials (225).

Besides, there are several studies investigating to increase the expression level of tumor-suppressive miRNA. Adeno-associated virus (AAV) mediated overexpression is one of the techniques used to increase the expression level of tumor-suppressive miRNA (178). In several types of cancer, such as BC, the reduced expression of miR-26a has been shown to be restored by overexpressing it in miRNA-based liver cancer models using AAV vectors. Another approach includes nanoparticles mediated overexpression, where the negatively charged miRNA molecules get transported using the positively charged nanoparticles (227). Nanoparticle mediated transfer confirms stability, prevention from nuclease degradation and increased efficiency (228). Shu et al. has demonstrated RNA nanotechnology-based delivery of anti-miR-21 resulting in decreased growth of TNBC in orthotopic mouse models at low doses (229). These studies suggest that miRNA-based techniques have the potential to be used as therapeutics in treating cancers including TNBC.

## 4 Conclusion

In this review we have explored the major subtypes of TNBC and the major genetic and epigenetic changes that give rise to TNBC. Also, we have discussed multiple therapeutic approaches to treat TNBC patients in detail, including histone deacetylase inhibitors (HDACi) as the main therapeutic target. HDACi trigger oxidative stress, promote DNA damage and interfere with apoptosis pathways to promote the expression of proapoptotic proteins. While HDACi alone may lack efficacy in inducing anti-tumor effect, its combination with kinase inhibitors have shown promising results by significantly reducing tumor burden and inhibiting cancer cell proliferation. Moreover, we provide insights into the possible miRNA involvement in TNBC as a tumor suppressor and oncogenic functionalized based on their modulating effects. Emerging miRNA therapeutics for cancer treatment are gaining prominence with novel strategies that warrant further validation through future experiments (118).
